# *N*-(4-iodophenyl)-*N*′-(2-chloroethyl)urea as a microtubule disrupter: *in vitro* and *in vivo* profiling of antitumoral activity on CT-26 murine colon carcinoma cell line cultured and grafted to mice

**DOI:** 10.1038/sj.bjc.6603778

**Published:** 2007-05-08

**Authors:** M Borel, F Degoul, Y Communal, E Mounetou, B Bouchon, R C-Gaudreault, J C Madelmont, E Miot-Noirault

**Affiliations:** 1 INSERM, U484, Clermont-Ferrand, F-63005 France; Université d'Auvergne, Clermont-Ferrand, F-63001 France; Centre Jean Perrin, Clermont-Ferrand, F-63011 France; 2 Unité des Biotechnologies et de Bioingénierie, Centre de recherche, CHUQ, Canada G1L 3L5

**Keywords:** tubulin, CT-26, colon carcinoma; uptake, phospholipids, ^1^H HRMAS NMR

## Abstract

The antitumoral profile of the microtubule disrupter *N*-(4-iodophenyl)-*N*′-(2-chloroethyl)urea (ICEU) was characterised *in vitro* and *in vivo* using the CT-26 colon carcinoma cell line, on the basis of the drug uptake by the cells, the modifications of cell cycle, and *β*-tubulin and lipid membrane profiles. *N*-(4-iodophenyl)-*N*′-(2-chloroethyl)urea exhibited a rapid and dose-dependent uptake by CT-26 cells suggesting its passive diffusion through the membranes. Intraperitoneally injected ICEU biodistributed into the grafted CT-26 tumour, resulting thus in a significant tumour growth inhibition (TGI). *N*-(4-iodophenyl)-*N*′-(2-chloroethyl)urea was also observed to accumulate within colon tissue. Tumour growth inhibition was associated with a slight increase in the number of G2 tetraploid tumour cells *in vivo*, whereas G2 blockage was more obvious *in vitro.* The phenotype of *β*-tubulin alkylation that was clearly demonstrated *in vitro* was undetectable *in vivo*. Nuclear magnetic resonance analysis showed that cells blocked in G2 phase underwent apoptosis, as confirmed by an increase in the methylene group resonance of mobile lipids, parallel to sub-G1 accumulation of the cells. *In vivo*, a decrease of the signals of both the phospholipid precursors and the products of membrane degradation occurred concomitantly with TGI. This multi-analysis established, at least partly, the ICEU activity profile, *in vitro and in vivo*, providing additional data in favour of ICEU as a tubulin-interacting drug accumulating within the intestinal tract. This may provide a starting point for researches for future efficacious tubulin-interacting drugs for the treatment of colorectal cancers.

The major role of microtubules in cell division makes tubulin one of the most prominent and studied target for the development of new antitumour drugs, especially for solid tumours. The taxanes and the vinca alkaloids are effective in the treatment of many cancers in humans, but their therapeutic efficacy is hampered by acquired or intrinsic drug resistance of the tumour cells (Cabral, 2001; Drukman and Kavallaris, 2002; Wilson and Jordan, 2004). To circumvent these problems, extensive research programmes have been focusing on finding selective therapies that are less prone to induce resistant phenotypes in tumour cells. In the past decade, *N*-aryl-*N*′-(2-chloroethyl)ureas (CEUs) have exhibited promising antitumoral activity through alkylation of *β*-tubulin near the colchicine-binding site. *N*-aryl-*N*′-(2-chloroethyl)ureas are molecular hybrids obtained from the biofunctional moiety of aromatic nitrogen mustards and the non-nitrosated pharmacophore of aliphatic nitrosoureas (Gaudreault *et al*, 1988; Lacroix *et al*, 1988; Bechard *et al*, 1994; Mounetou *et al*, 2001, 2003). *N*-(4-iodophenyl)-*N*′-(2-chloroethyl)-urea (ICEU) was selected for study on the basis of a low inhibition concentration (IC_50_) on several tumour cell lines resistant to numerous other chemotherapeutic agents. *In vitro* studies have shown that ICEU cytotoxicity is linked to cytoskeleton disruption following microtubule depolymerisation, resulting from *β*-tubulin alkylation of the glutamic acid residue at position 198 (Petitclerc *et al*, 2004; Bouchon *et al*, 2005). Finally, ICEU has displayed significant antitumoral activity in the murine CT-26 colon carcinoma model, antitumoral activity that is, at least partly, associated with an antiangiogenic effect (Miot-Noirault *et al*, 2004; Petitclerc *et al*, 2004). Furthermore, ICEU can be readily radiolabelled with either iodine-123 (^123^I) for scintigraphic analysis or iodine-131 (^131^I) for radiotherapy applications.

The purpose of the study was to characterise the antitumoral profile of ICEU using four main criteria. Using the CT-26 murine colon carcinoma cell line, we first studied ICEU uptake and its effects on the cell cycle, *β*-tubulin and membrane lipid profiles *in vitro*; afterwards, we tried to correlate *in vitro* findings with those on CT-26 tumours grafted to mice. The data showed that [^125^I]ICEU was rapidly absorbed and distributed to the CT-26 colon carcinoma tissue *in vivo* and being taken up by the cells according to a passive diffusion mechanism. *N*-(4-iodophenyl)-*N*′-(2-chloroethyl)urea induced inhibition of cell growth through arrest of cell division in G2 phase in either CT-26 cells or in CT-26-derived tumours. Sodium dodecyl sulphate (SDS)-PAGE analysis of ICEU-treated cells and tumours showed clearly the phenotype of *β*-tubulin alkylation *in vitro*, but not *in vivo*. Furthermore, proton high-resolution magic angle spinning nuclear magnetic resonance (^1^H-HRMAS NMR) analysis showed evidence of changes in both the mobile lipids and the phospholipids, those changes being characteristic of the apoptosis taking place *in vitro* and the reduction of cell membrane turnover and the cell proliferation occurring *in vivo*.

This multi-analysis brings additional data that contribute to a better understanding of the antitumoral action of ICEU.

## MATERIALS AND METHODS

### CT-26 murine carcinoma cell line

The CT-26 cell line was kindly provided by Dr IJ Fidler (MD Anderson Cancer Center, Houston, TX, USA).

For *in vitro* experiments, cells were grown as monolayer cultures in minimum essential medium with Earle's salts (MEM®, Gibco-BRL, Cergy Pontoise, France) supplemented with 10% foetal calf serum (Sigma-Aldrich, St Quentin, Fallavier, France), 1% solution of vitamins (Gibco-BRL), 1% sodium pyruvate (Gibco-BRL), 1% nonessential amino acids (Gibco-BRL) and 0.04% gentamycin base (Gibco-BRL) at 37°C in 5% CO_2_ and at 100% humidity.

For *in vivo* experiments, CT-26 tumour grafts were established in 67 male 6-week-old Balb/c mice (Charles River, L'Arbresle, France) by subcutaneous (s.c.) injection of 2 × 10^5^ CT-26 cells in the right flank at day 0. Animals were randomly assigned to either a biodistribution study (*n*=42) or an antitumoral efficacy study (*n*=25). Tumour volumes (in mm^3^) were calculated from the measurement of two perpendicular diameters using a caliper, according to the formula: *L* × *S*^2^/2, where *L* and *S* are the largest and smallest diameters in mm, respectively.

All animal studies were performed under the authorisation of the French Veterinary Services Directorate (authorisation no. C63-113-10) and conducted under the supervision of authorised investigators in accordance to the institution's recommendations for the use of laboratory animals and with UKCCCR guidelines (Workman *et al*, 1998).

### *In vitro* and *in vivo* drug treatment

**Figure 1:**
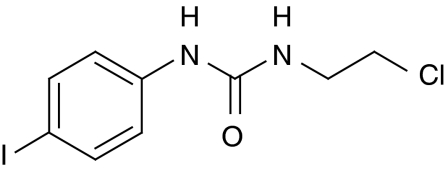
Chemical structure of *N*-(4-iodophenyl)-*N*′-(2-chloroethyl)urea (ICEU).

*N*-(4-iodophenyl)-*N*′-(2-chloroethyl)urea was synthesised (Figure 1) as previously described (Mounetou *et al*, 2001).

For *in vitro* experiments, both unlabelled ICEU and [^125^I]ICEU (specific activity: 1.5 GBq mmol^−1^) were dissolved in dimethylsulphoxide (DMSO). Two million (2 × 10^6^) cells were plated in 10-mm Petri dishes 24 h prior to the addition of escalating concentrations of the drug for different period of time. After incubation, the cells were harvested by scraping. The cell suspension containing both floating and adherent cells was spun twice (400 **g**, 8 min, 4°C) in PBS. Dry pellets were then stored (i) in liquid nitrogen prior to cell cycle and western blotting analyses or (ii) at −80°C until ^1^H-HRMAS NMR analysis.

For the cellular uptake study, after incubation with [^125^I]ICEU, the medium was quickly removed and the cells were washed with cold PBS, scraped and counted for radioactivity (Minaxi g 5530 gamma counter, Packard Rungis, Rungis, France). Drug uptake was expressed in pmoles of [^125^I]ICEU per *μ*g of proteins, with the protein content being determined by the Bradford's method using Coomassie Blue G250 solution (Pierce, Oud-Bejerland, The Netherlands) (Bradford, 1976; Sapan *et al*, 1999).

For *in viv*o experiments, both unlabelled ICEU and [^125^I]ICEU (specific activity=734.5 MBq mmol^−1^) were dissolved in a mixture of Labrafils® M1944 Cs (Gattefossé, Gennevilliers, France), dimethylacetamide (Sigma-Aldrich, France), and Tween 80® (Sigma-Aldrich, France) (89/9/1%, v v^−1^) as previously described (Miot-Noirault *et al*, 2004). For biodistribution studies, the previously determined maximum-tolerated dose (MTD) of 13 mg kg^−1^ (Miot-Noirault *et al*, 2004) of [^125^I]ICEU (activity per mouse: 74 KBq) were administered intraperitoneally (i.p.) to the animals when tumours had reached approximately 5 mm in diameter (day 10).

For the antitumoral efficacy study, we used the previously published ICEU ‘infraclinical protocol’, since it was successful in inducing antitumour effects similar to those observed when using 5-fluorouracil (5-Fu) as positive control (Miot-Noirault *et al*, 2004; Petitclerc *et al*, 2004). *N*-(4-iodophenyl)-*N*′-(2-chloroethyl)urea was therefore injected i.p at the MTD of 13 mg kg^−1^ at days 1, 5 and 9 (*n*=13 animals), while mice in the control group (*n*=12) were sham treated. During this antitumoral efficacy study, the toxic effects of ICEU were assessed by the evaluation of the ‘clinical state’ of each animal where both the mortality and ‘poor health’ symptoms such as weight loss, lethargy, rough coat, closed eyes and diarrhoea were assessed.

Our objective was also to compare ICEU-treated and control tumour samples for cell cycle, *β* tubulin and lipid profiling, when a high growth inhibition was observed. Two tumours from control and treated groups were excised at days 9, 11, 15 and 18, on the basis on our previous results that have demonstrated a high inhibition level of the tumour growth at the stages day 9 and day 11 and a growth relapse from day 15 (Miot-Noirault *et al*, 2004). The tumours were carefully divided in three equivalent aliquots that were immediately and respectively disaggregated for cell cycle analysis, frozen in liquid nitrogen, or stored at 80°C until western blotting or analysed by ^1^H HRMAS NMR spectroscopy.

### Tissue distribution of [^125^I]ICEU in CT-26-bearing mice (*n*=42)

At different time points after administration, the animals (*n*=30; 3 animals per time point) were killed by CO_2_ inhalation, and then immediately frozen in liquid nitrogen. The frozen animals were cryosectioned into 40 *μ*m slices, and the radioactivity content of the samples was assessed using quantitative whole body autoradiography (WBA) as previously described (Labarre *et al*, 1999). Since WBA evaluation of colon radioactivity included the colon mucosa and its contents, the radioactivity of the colon alone was assessed from washed colon tissue aliquots excised from additional CT-26-bearing mice (*n*=12) killed at the same time points, using a radioactivity counter (Minaxi *γ* 5530, Packard, Rungis, France). Uptake values were corrected for radioactive decay and expressed as percentage of the injected dose per gram of tissues (% ID g^−1^).

### Flow cytometry DNA analysis

CT-26 tumour samples were mechanically disaggregated in PBS solution by fine mincing with 26G needles and filtering through a 200 *μ*m nylon filter. The suspensions of cells were spun (400 **g**, 8 min, 4°C), and the dry pellets obtained were stored in liquid nitrogen. After thawing, the cell pellets and the tumour cell extracts were resuspended in 500 *μ*l of ribonuclease A (1 mg ml^−1^) (Sigma-Aldrich, France) and 500 *μ*l of propidium iodide (1 mg ml^−1^) (Sigma-Aldrich, France) was added. After 30 min at 4°C in the dark, the cell cycle was analysed using a flow cytometer (Coulter Epics XL, Coulter, Hialeah, FL, USA) at wavelengths of 488 and 620 nm, respectively.

### *β*-Tubulin western blot analysis

For cell extracts, pellets were directly resuspended in Laemmli buffer 1 × as previously described (Legault *et al*, 2000). For tumour cell extracts, samples were crushed and homogenised in one volume of 10 mM Tris-HCl buffer at pH 7.5 containing 0.25 M sucrose and 10 mM ethylenediaminetetraacetic acid, and 4 volumes of the solubilisation buffer containing 8.4 M urea, 2.4 M thiourea, 50 mM dithiothreitol and 5% 3-[(3-cholamidopropyl)dimethylammonio]-1-propanesulphonic acid (CHAPS) with a cocktail of protease inhibitors. Extraction was carried out for 30 min at room temperature by vigorous shaking of the mixture and was followed by ultracentrifugation at 100 000 **g** for 30 min. The supernatants were recovered and the protein content was determined by the Bradford's method as described above.

Sodium dodecyl sulphate-PAGE electrophoresis was performed on a 10% acrylamide gel and the proteins were transferred onto nitrocellulose membranes (Immobilon NC, Millipore, St-Quentin-en Yvelines, France). Membranes were incubated with an anti-*β*-tubulin anti-mouse antibody (Anti TUB2.1, Sigma, St-Quentin-Fallavier, France). An additional incubation was carried out with an HRP-labelled anti-mouse antibody (Dako, Trappes, France) to reveal *β*-tubulin by chemoluminescence.

For cells treated with [^125^I]ICEU, protein extracts were processed as described above and autoradiographic analysis was performed by exposing the dried gels to films.

### The ^1^H-HRMAS NMR spectroscopy and data analysis

Proton high-resolution magic angle spinning nuclear magnetic resonance spectroscopy was used to investigate the lipid profile of both CT-26 cells and tumours after ICEU treatment.

This technique consists of spinning the sample at a rate <10 kHz around an axis tilted at 54.7° (magic angle) from the magnet axis. The combination of a high-field magnet with the HRMAS accessory allowed us to obtain high-resolution spectra of small amounts (0.5–1 mg) of biological materials. Analyses were performed on a small-bore Bruker DRX 500 magnet (Bruker, Karlsruhe, Germany) equipped with an HRMAS accessory. CT-26 cell preparations (8 × 10^6^ cells in saline deuterium 9/1000 solution) or intact CT-26 tumour material were set into 4-mm-diameter, 80-*μ*l Zirconia rotor tubes. One-dimensional (1D) ^1^H NMR spectra were acquired at the magic angle at 293 K with a rotor spin of 4 KHz using 90° pulses preceded by 2 s presaturation for water signal suppression, giving a total measurement time of 48 min for 1024 scans. For tumour sample analysis, 1D NMR spectra were completed by two-dimensional homonuclear correlated spectra (2D COSY) recorded by acquisition of 16 transients per increment for 512 increments collected into 1 K data points. A spectral width of 5 kHz was used in both dimensions. The time-domain data were zero-filled and multiplied with a sine window function in both dimensions before Fourier transformation. For data analysis, each spectrum was phased and baseline-corrected using XWIN-NMR version 2.6 (Bruker software). The resonances were assigned based on the known chemical shifts of the major structural groups and metabolites using published data (Bezabeh *et al*, 2001; Sitter *et al*, 2002; Brisdelli *et al*, 2003) and commercially available pure metabolite reference spectra. The relative content of metabolites was estimated by peak area integration using PeakFit software (version 4, SPSS Science, Chicago, IL, USA). The intensity of the contribution of *β-* and *δ*-methylene protons of lysine (lys) to the signal at 1.71 ppm remained invariant during cell and tumour sample manipulation, hence, the deconvolution area of the lys signals was used as a reference as described previously (Brisdelli *et al*, 2003). To compare the metabolic profiles between experimental groups, metabolic ratios were calculated by dividing the integrated area under the curve of each relevant peak by the integrated area under the curve of the reference peak.

Tumour and cell sample stability inside the rotor was assessed over several hours by a series of analyses. During the 48-min overall acquisition period, no significant changes in NMR signals of biological material were observed.

Each experiment was performed in triplicate.

### Statistics

Data on tumour volumes and data from NMR analyses were compared between control and treated groups using the Student's *t*-test for independent means. Two-sided *P*-values less than 0.05 was considered significant.

## RESULTS

### *N*-(4-iodophenyl)-*N*′-(2-chloroethyl)urea induced growth inhibition of CT-26 cells grafted to mice

**Figure 2:**
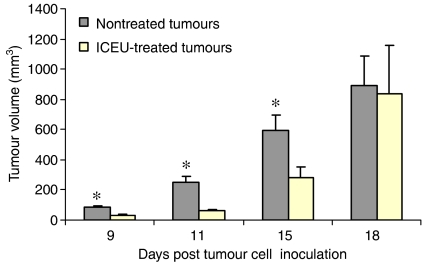
*N*-(4-iodophenyl)-*N*′-(2-chloroethyl)urea delayed the growth of CT-26 colon carcinoma tumours in mice. Effect on tumour progression of ICEU administered i.p. (13 mg kg^−1^) at days 1, 5 and 9 after tumour cell inoculation (12 animals per group). Data correspond to average values of tumoral volume with standard deviations indicated. Tumour growth inhibition was statistically significant at days 9, 11 and 15.

In the antitumour efficacy experiments, mice received three intraperitoneal (i.p.) injections of ICEU (13 mg kg^−1^) at 4-day intervals, starting on day 1 after cell inoculation (Figure 2). Mean tumour volume was significantly lowered in the ICEU-treated group than in the control group at day 9 (33.5±7.8 *vs* 83.4±7.4 mm^3^), day 11 (61.3±12.1 *vs* 249.2±42.7 mm^3^) and day 15 (283.5±68.1 *vs* 594.8±99.4 mm^3^), corresponding to a tumour growth inhibition of 59.8, 75.4 and 52.3% respectively. From the stage day 15, the ICEU-treated tumours were observed to relapse, with no significant differences between control and treated groups being observed at day 18. During the treatment of mice with ICEU using the ‘infraclinical protocol’, that is, ICEU given at the MTD of 13 mk kg^−1^ injection^−1^ at days 1, 5 and 9 after tumour cell inoculation, no major toxicity was observed. It is of interest to mention that some signs of rough coat appeared in 100% of the animals and that lethargy and closed eyes were observed for 25% of the mice. Maximal weight loss was 4% observed at day 11, that is, 2 days after the last administration of ICEU.

### *N*-(4-iodophenyl)-*N*′-(2-chloroethyl)urea distributed to CT-26 tumour *in vivo* and accumulated in CT-26 cells *in vitro*

**Figure 3:**
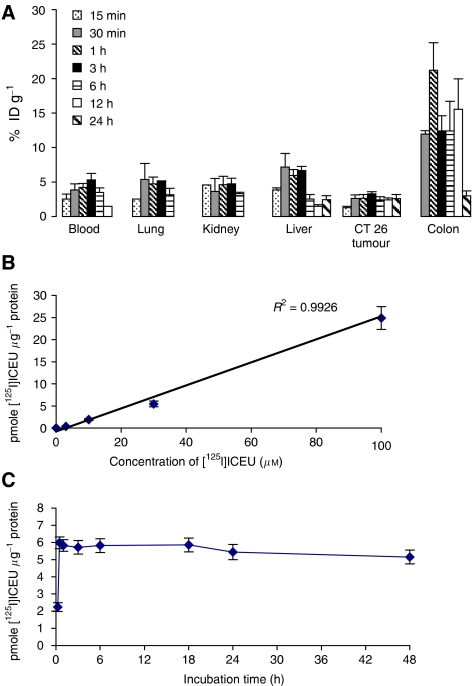
*N*-(4-iodophenyl)-*N*′-(2-chloroethyl)urea distributed to tumour cells *in vitro* and *in vivo*. (**A**) [^125^I]ICEU distribution in CT-26 tumour graft and tissues of mice. [^125^I]ICEU (74 kBq) was administered i.p. to mice bearing CT-26 tumours. Data correspond to average uptake values of [^125^I]ICEU (in % ID g^−1^), and bars illustrate s.e.m. (three mice per time point). Radioactivity was retained within CT-26 tumours (3.3% ID g^−1^) and the colon (15% ID g^−1^). (**B**) [^125^I]ICEU uptake by CT-26 cells as a function of drug concentration in the extracellular medium. Data correspond to average uptake values with standard deviations indicated (experiments were performed three times). [^125^I]-ICEU uptake was dose dependent. (**C**) Kinetics of [^125^I]ICEU (30 *μ*M) uptake by CT-26 cells over a 48-h period. Data correspond to average values with standard deviations indicated (experiments were performed three times). Intracellular concentrations reached the plateau after 1 h of exposure.

To determine both the intratumoral uptake of the drug and its biodistribution pattern, CT-26 tumour-bearing mice (mean tumour volume=176.9±29.8 mm^3^) were treated i.p. with [^125^I]ICEU at the MTD (13 mg kg^−1^; 74 kBq). As early as 15 min postinjection, radioactivity was largely detected in the blood circulation and in well-vascularised organs such as the lungs, liver and kidneys, confirming the absorption of the drug (Figure 3A). Tumour uptake of the radioactivity was also observed 15 min after administration with 1.3±0.1% of ID g^−1^, reaching a stable maximal value of 3.3±0.3% ID g^−1^ at 3 h until 24 h postinjection. Interestingly, radioactivity levels remained stable within the tumour from 3 h after injection while it was eliminated from blood and others organs. We also observed an unexpected high accumulation of radioactivity within the intestinal tract. To determine whether this radioactive signal was coming from the intestinal content or from the colonic tissue itself, we performed radioactive counting of excised and washed colon tissue aliquots. This additional study confirmed a high level of radioactivity accumulation (reaching about 15% ID g^−1^ from 30 min to 12 h postadministration) within the intestinal tissue.

More than 75% of the dose of ICEU injected was eliminated during the first 48 h, with 53.3% of the dose being excreted in urine (data not shown).

We have also evaluated the *in vitro* accumulation of [^125^I]ICEU in CT-26 cells after 24 h of drug incubation at concentrations ranging from 3 to 100 *μ*M. As illustrated in Figure 3B, [^125^I]ICEU uptake was dose dependent (*R*^2^=0.9926). Concerning the kinetics of [^125^I]ICEU uptake by the cells, uptake was rapid with intracellular concentrations plateaued after 1 h of incubation (Figure 3C). Maximal [^125^I]ICEU uptake was 5.8 pmoles drug *μ*g^−1^ proteins (i.e. 9.2% of incubated dose) from 1 to 18 h of incubation. From 24 to 48 h of incubation, [^125^I]ICEU uptake fell to 5.1 pmoles drug *μ*g^−1^ proteins, but the decrease was not significant.

### *N*-(4-iodophenyl)-*N*′-(2-chloroethyl)urea induced cell cycle modifications *in vitro* and *in vivo*

**Figure 4:**
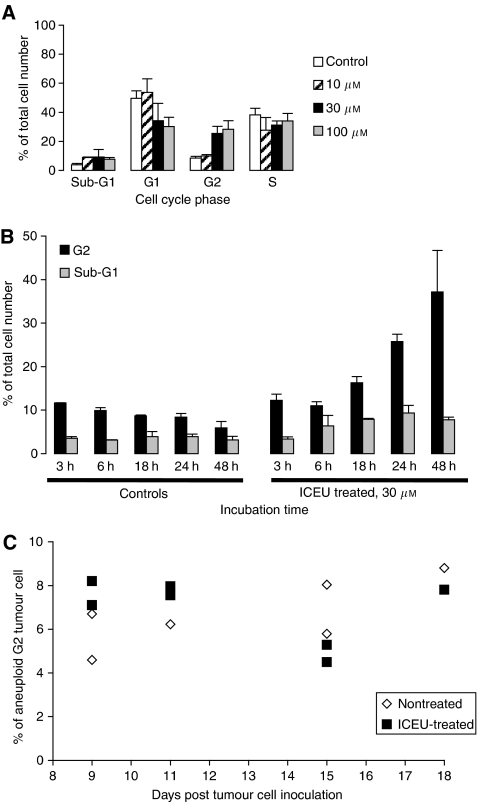
*N*-(4-iodophenyl)-*N*′-(2-chloroethyl)urea induced perturbations in cell cycle distribution in CT-26 colon carcinoma cells, both *in vitro* and *in vivo*. Data correspond to average values with standard deviations indicated (experiments were performed three times). (**A**) Cell cycle distribution as a function of ICEU doses. *N*-(4-iodophenyl)-*N*′-(2-chloroethyl)urea treatment induced a significant accumulation of cells in both the G2 phase and the sub-G1 phase. (**B**) Kinetics of G2 and sub-G1 accumulation in ICEU-treated cells. G2 and sub-G1 populations increased exponentially with time. (**C**) Distribution of cells extracted from both control and ICEU-treated tumours in the aneuploid G2 phase. At days 9 and 11, there was a slight increase in the aneuploid G2 population in treated samples compared to controls.

In controls, 38.2±4.6% of CT-26 cells accumulated in the S phase, thus reflecting a high proliferative activity. *N*-(4-iodophenyl)-*N*′-(2-chloroethyl)urea treatment led to an increase in cell accumulation in the G2 and sub-G1 phases (Figure 4A). Compared to controls, escalating doses of ICEU induced a 1.2–4-fold increase of the G2 population and a twofold increase of the sub-G1 content, which is indicative of apoptosis. We studied the kinetics of this response and observed that G2 and sub-G1 populations increased exponentially with drug exposure. Nevertheless, both G2 and sub-G1 populations were significantly increased as compared to controls after 18 h of drug exposure (Figure 4B).

Both control and ICEU-treated CT-26 tumours exhibited an aneuploid cellular population in addition to the diploid population (data not shown). Since G1 and sub-G1 aneuploid peaks were difficult to distinguish from the G2 diploid peak, we only took into account aneuploid G2 distribution. Figure 4C shows individual percentages of accumulated aneuploid cells in G2 phase for both control and ICEU-treated tumours at several time points during therapy. At days 9 and 11, we observed a slightly higher aneuploid G2 population in treated samples than in controls. This trend was reversed from day 15 when tumour growth relapsed.

### *N*-(4-iodophenyl)-*N*′-(2-chloroethyl)urea induced alterations in *β*-tubulin migration on electrophoresis that were detected *in vitro* but not *in vivo*

**Figure 5:**
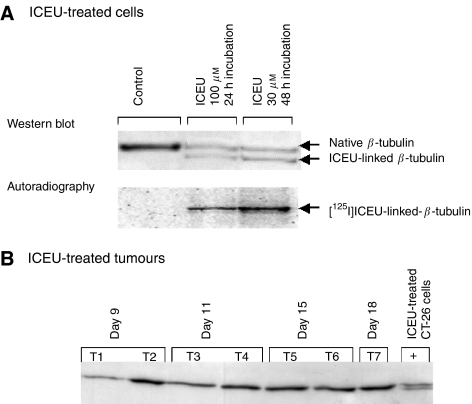
*N*-(4-iodophenyl)-*N*′-(2-chloroethyl)urea-induced alterations in *β*-tubulin migration properties on SDS–PAGE gel; alterations were detected *in vitro* but not *in vivo*. (**A**) Western blotting of *β*-tubulin from extracts of CT-26 cells incubated with either DMSO or ICEU (unlabelled or labelled with ^125^I): The alkylated *β*-tubulin migrated faster than the native form (upper panel). This additional immunoreactive band could be superimposed over the radioactive band, as shown by autoradiography (lower panel). (**B**) Western blotting of *β*-tubulin from CT-26 tumour extracts. Tumours were sampled at day 9 (samples T1 and T2), 11 (samples T3 and T4), -15 (samples T5 and T6) and -18 (sample T7) after tumour cell inoculation. The additional immunoreactive band of *β*-tubulin was not detected. Western blotting of ICEU-treated CT-26 cell extract (noted +) was added.

Alkylation of *β*-tubulin by CEUs has been shown to induce a change in the *β*-tubulin migration behaviour. We looked for this phenotype in cells incubated with labelled or unlabelled ICEU (Figure 5A). CT-26 cells incubated with 100 *μ*M [^125^I]ICEU for 24 h displayed a double immunoreactive band of *β*-tubulin (upper panel, Figure 5A) that could be superimposed over the radioactive ICEU protein (lower panel, Figure 5A); the ICEU-induced alkylation of *β*-tubulin occurred in cultured CT-26 cells.

We then looked for the ‘*β*-tubulin double band phenotype’ on western blots of ICEU-treated tumours at several stages, that is, (i) days 9 and 11 that corresponded to a high tumour growth inhibition, (ii) day 15 to a moderate tumour growth inhibition and (iii) day 18 that corresponded to tumour relapse. The *β*-tubulin alkylation phenotype could not be revealed on any samples (Figure 5B). Nevertheless, the intensity of the *β*-tubulin band appeared to be lower at days 9 and 11, which could be correlated to a lower protein content (*β*-tubulin/*β*-actin ratio lower at these two stages only, data not shown).

### *N*-(4-iodophenyl)-*N*′-(2-chloroethyl)urea induced changes in phospholipid membrane profile both *in vitro* and *in vivo*

**Figure 6:**
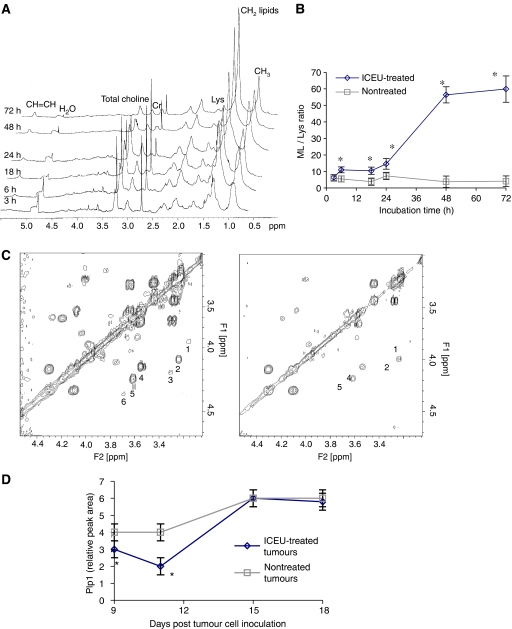
Proton high-resolution magic angle spinning nuclear magnetic resonance analysis of CT-26 cells and tumours treated with ICEU. (**A**) Time-dependent ^1^H spectra changes in ICEU-treated cells. ^1^H (500 MHz, 512 averages) spectra from bottom to top correspond to CT-26 cells incubated with ICEU (30 *μ*M) for 3, 6, 18, 24, 48 and 72 h. An increase in the signal intensity of both the mobile lipids and the polyunsaturated fatty acids, and a decrease in the signal intensity of choline were observed. (**B**) Mobile lipids/lys ratio of both untreated and ICEU-treated cells as a function of drug incubation time. Data correspond to average values with standard deviations (experiments were performed three times). In ICEU-treated cells, there was an increase in ML/lys ratio that followed a ‘two-step kinetics’ pattern, that is at an early (3–6 h after drug exposure) and then at a late stage (18–72 h after drug exposure). (**C**) Two-dimensional homonuclear correlated spectra COSY ^1^H spectra of both untreated (on the left) and ICEU-treated tumours (on the right-hand side) at day 11 (CT-26-bearing mice received ICEU (13 mg kg^−1^) by i.p. route at days 1, 5 and 9 postimplantation). Cross peak assignments: (1) ethanolamine (2) phosphoethanolamine (3) glycerophosphoethanolamine (4) choline (5) phosphocholine and (6) glycerophosphocholine. (**D**) Relative peak area of Plp1 of both untreated and ICEU-treated tumours as a function of time during therapy. CT-26-bearing mice received ICEU (13 mg kg^−1^) by i.p. route at days 1, 5 and 9 postimplantation. Plp1 decreased significantly in ICEU-treated tumours at days 9 and 11 compared to controls.

Typical 1D ^1^H-NMR spectra of untreated and ICEU-treated CT-26 cells showed well-resolved resonances in the spectral region of 0.8–5.2 ppm (Figure 6A): the predominant peaks arose from fatty acyl chains of lipids thus called mobile lipids (ML), with the resonances of the methyl groups (CH_3_) and the methylene groups (CH_2_) observed at 0.85–0.90 and 1.20–1.32 ppm, respectively, the doublet of lactate at 1.32 and 1.34 ppm, lys at 1.71 ppm, creatine at 3.03 ppm, choline-containing compounds between 3.20 and 3.23 ppm, and resonances from polyunsaturated fatty acids (PUFA) at 5.20 ppm. *N*-(4-iodophenyl)-*N*′-(2-chloroethyl)urea treatment of CT26 cells led to changes in the signal intensity of ML, PUFA and choline as a function of the period of exposure to the drug (Figure 6A). For each incubation time, quantitative analysis focused on the changes in ML/lys ratio respectively to controls. As shown in Figure 6B, after 6 h of incubation, the mean ML/lys ratios were significantly higher in ICEU-treated cells than in controls. It should be noted that ICEU-treated cells showed two ‘waves’ of ML/lys ratio increase. In one hand, a first ‘wave’ occurred between 3 and 6 h (ML/lys=10.96±1.8 for ICEU-treated cells *vs* 5.46±1.8 for controls at the 6 h time point). On the other hand and after an intermediate plateau (6–18 h), a second sharp strong increase in ML/lys ratio was observed between 24 and 72 h time, reaching peak ML/lys ratio values of 59.9±8 for treated cells *vs* 4.1±3 for controls at the 72 h time point. To perform assignments of the ML involved in this signal, 1D ^31^P spectra of chloroform/methanol extracts were performed for both untreated and ICEU-treated CT-26 cells. The results were then compared for phospholipid metabolic changes. The phosphatidylethanolamine to phosphatidylcholine ratio was significantly higher in ICEU-treated cells (0.4±0.03) than controls (0.25±0.02) (data not shown).

The antitumoral activity of ICEU was also investigated, *in vivo* by comparing treated tumour samples to controls at different time points. As observed with the cell lines, resonances of methylene groups from ML (1.20–1.32 ppm) and PUFA (5.20 ppm) of ICEU-treated tumours increased in a time-dependent manner relatively to lys, but the variation was weaker than *in vitro* (data not shown). The most prominent changes in ICEU-treated tumour samples were observed from signals acquired by ^1^H 2D COSY spectra (Figure 6C) that distinguished the signals from choline (3.55 ppm), ethanolamine (3.15 ppm), phosphocholine (3.62 ppm), phosphoethanolamine (3.22 ppm, both were designated as Plp1) and peaks deriving from glycerophosphocholine (GPC) (3.68 ppm) and glycerophosphoethanolamine (GPE) (3.30 ppm, both were named as Plp2), known to be related to phospholipid biosynthesis and breakdown *in vivo*, respectively. Figure 6C illustrates the typical ^1^H 2D COSY spectra of both the control and the treated tumours at day 11 where a significant decrease of the metabolite signals could be attributed to ICEU treatment, especially for peaks deriving from Plp1 and Plp2. The changes in the relative peak areas for both Plp1 and Plp2 were analysed in treated tumours and compared to controls. From day 9 (3 h after the last treatment) to day 18 (9 days after the last treatment), Plp2 signal decreased to very low levels that were indistinguishable of the NMR background and did not allow any quantitative analysis. For Plp1 signal, a significant decrease in ICEU-treated tumours compared to controls was observed at days 9 and 11 (Figure 6D). From day 15, there were no significant differences in Plp 1 between ICEU-treated tumours and controls.

## DISCUSSION

The aim of this study was to investigate the antitumoral activity of the new tubulin-interacting drug ICEU in the murine CT-26 colon carcinoma cell line at both the tissues and the cellular level. After i.p. administration, ICEU was rapidly absorbed and largely distributed in well-vascularised organs. Tumour uptake of [^125^I]ICEU reached a stable maximal value of 3.3±0.3% ID g^−1^. To our knowledge, this level of drug accumulation in the tumour appears higher than that of 5-Fu accumulation, that is the current ‘gold standard’ drug for the first-line of colorectal cancer treatment, reported in the same CT-26 tumour-bearing mice model (Kamm *et al*, 2003). These data appeared to us of particular interest since they supported our previous findings demonstrating that 40 *μ*mol kg^−1^ of ICEU administered i.p. was equipotent to 150 *μ*mol kg^−1^ of 5-Fu administered i.v. to inhibit the growth of CT-26 colon carcinoma tumours and increased lifespan of the animals (Miot-Noirault *et al*, 2004). Interestingly, [^125^I]ICEU was retained within the tumour for a long time period while it was eliminated from most organs 6 h post administration. This observation may act in favour of the *in vivo* antitumour activity of ICEU since it appears from cell cycle kinetics analysis that a long ICEU exposure time is necessary to induce a G2 blockage. It seemed therefore essential to determine whether the long exposure time necessary to observe a cytotoxicity was attributable to a difficulty for the drug to reach the cells or was inherent to its mechanism of action. Results from the cellular uptake study evidenced a rapid dose-dependent uptake of ICEU by CT-26 cells. This suggested a passive diffusion of the drug throughout the biological membranes, as described previously for many other lipophilic tubulin-interacting agents (Singer and Himes, 1992). To that end, ICEU exhibits a calculated Log *P* of 2.8 (ChemDraw software) and has been shown to interact easily with model lipid bilayer membranes (Saint-Laurent *et al*, 2001). Considering the cytotoxic activity of ICEU, it was recently demonstrated on B16 melanoma cells that the mechanism of action of ICEU was mediated through its covalent binding to a glutamic acid residue at position 198 of *β*-tubulin isoform 5 (Bouchon *et al*, 2005). In CT-26 cells, ICEU induced similar modifications of the *β*-tubulin migration properties on SDS–PAGE. ICEU-modified-*β*-tubulin and labelled ICEU proteins could be superimposed, suggesting that the additional *β*-tubulin band results from the irreversible binding of ICEU to the protein. We also looked for the ‘*β*-tubulin double band phenotype’ on western blots of ICEU-treated tumours at several stages during and after the treatment, that is, days 9, 11, 15 and 18 of the protocol. This sampling time window was chosen at several stages of the CT-26 tumour response to ICEU treatment on the basis of our previously published results that have shown with the same protocol, (i) a high inhibition of the tumour growth at the stages day 9 and day 11, (ii) a moderate growth inhibition at day 15 and (iii) a relapse of the tumours at day 18 (Miot-Noirault *et al*, 2004). No *β*-tubulin alkylation phenotype could be observed, even for the stages day 9 and day 11, which corresponded to a tumour growth inhibition of 59.8 and 75.4%, respectively. Nevertheless, at these two stages, some ICEU-treated tumours showed a low *β*-tubulin/*β*-actin ratio, thus suggesting a lower content of native *β*-tubulin. We were unable to detect *in vivo* the additional band corresponding to the alkylated tubulin: this was a typical case of lack of correlation between a drug's cellular effects and its *in vivo* antitumour effect. That phenomenon is not unique, as it has been already reported for other antimicrotubule agents by Schimming *et al* (1999). We hypothesise that cells with alkylated *β*-tubulin might be eliminated quickly *in vivo* via phagocytosis and/or ubiquitin proteasome degradation pathways, as described previously (Krysko *et al*, 2006; Liu *et al*, 2006; Wang *et al*, 2006). Likewise, the tumoricidal concentration of ICEU necessary to kill cells should be higher as compared to *in vitro* when one considers the interrelated influence of physiological parameters such as perfusion, necrosis, cell density and interstitial pressure that may affect the diffusion of the drug into the tumoral tissues and the neoplastic cells. This difference *in vitro vs in vivo* was also observed with data obtained from cell cycle experiment analysis: *In vitro*, a percentage (around 40% of total cell number) were blocked in G2 phase while *in vivo* only a slight increase in the G2 tetraploid phase could be observed at days 9 and 11, which are both corresponding to the significant tumour growth inhibition observed. After day 15, the aneuploid G2 population was lower in treated samples than in controls. As previously hypothesised, the tumour cells that exhibited an aneuploid status (due to a block in mitosis by ICEU treatment) probably underwent apoptosis and were quickly eliminated via immune system (Weaver and Cleveland, 2005). We have monitored the ‘death programme’ (i.e. apoptosis or cell necrosis) of CT-26 cells cultured and grafted to mice, when exposed to ICEU. Toward that end, ^1^H-NMR spectrometric analysis was used as a quantitative method for the noninvasive evaluation of biochemical processes involved in cell proliferation and growth arrest (Valonen *et al*, 2005). Moreover, we performed the ^1^H-NMR analysis at the magic angle, which is called ^1^H-HRMAS NMR spectroscopy, that provides an improved spectral resolution and allows a direct analysis of ‘semisolid’ samples (cell pellet or tumour tissue) without the need of tissue extraction procedures. Since ^1^H-HRMAS NMR analysis do not compromise the biochemical and structural integrity of cells and tissues, it is considered that the lipid changes detected by a such methodology originate from the biochemically active pools of the sample (Weybright *et al*, 1998). From our results, we may hypothesise that cells blocked in G2 phase underwent apoptosis, as confirmed by a sharp increase in CH_2_ of ML resonances in NMR spectra: *In vitro*, when CT-26 cells were exposed to ICEU, a sharp increase in CH_2_ of ML resonances was observed. This lipid signal arose from fatty acyl chains in triglycerides arranged in microdomains embedded in the plasma membrane bilayer (Blankenberg *et al*, 1996, 1997). Interestingly, in ICEU-treated CT-26 cells, ML were generated at an early stage (3–6 h) as well as a late stage (18–72 h) after drug exposure. The early lipid signal changes probably arose from the interactions described recently and occurring between ICEU and the lipid bilayer membranes (Saint-Laurent *et al*, 2001). The late lipid signal changes (observed after 18 h) may be considered as markers of the apoptotic cascade, therefore reflecting plasma membrane reorganisation and phosphatidylserine externalisation (Blankenberg *et al*, 1996, 1997; Bezabeh *et al*, 2001; Brisdelli *et al*, 2003). Moreover, after the latter stage, there was a decrease in choline that indicates cell death (Blankenberg *et al*, 1996, 1997). In ICEU-treated tumours, there was a decrease in the Plp1 membrane precursors detected (i.e. phosphocholine and phosphoethanolamine), suggesting that ICEU causes a reduction in cell membrane turnover and cell proliferation, thus leading to the decrease of Plp 2 membrane degradation products (GPC and GPE) simultaneously to tumour growth inhibition (Lindskog *et al*, 2004; Valonen *et al*, 2005). When tumour proliferation relapsed (as confirmed by tumour volume assessment), there were no differences in phosphoethanolamine and phosphocholine levels between treated and control tumours. Despite tumour growth recovery, Plp 2 signal remained at low levels. This may be attributed to a certain level of necrosis in the tumour as a consequence of an antiangiogenic activity as commonly described for anti-tubulin agents (McPhail *et al*, 2005). It is noteworthy that ICEU has been described recently to block angiogenesis (Petitclerc *et al*, 2004).

In summary, ICEU is readily absorbed and rapidly biodistributed to CT-26 carcinoma tumour tissues and cells, inducing *β*-tubulin alkylation that leads to a G2 phase arrest, inhibition of cell proliferation and a reduction in cell membrane turnover and proliferation, events that all together contributed to tumour growth inhibition. Moreover, the treatment appeared ‘well-tolerated’ with no major side effects being observed. Unexpectedly, a high accumulation of radioactivity was observed within the intestinal tract, which was attributed to colon tissue structure. To our opinion, the accumulation of [^125^I]ICEU within colon tissues could be an important feature for further investigations with a view of developing efficacious treatments for colorectal cancers. In the light of these results, research is in progress to assess both the distribution and efficacy of ICEU in the CT-26 tumour, being orthotopically implanted in mice.
